# Double-stranded RNA orbivirus disrupts the DNA-sensing cGAS-sting axis to prevent type I IFN induction

**DOI:** 10.1007/s00018-025-05580-5

**Published:** 2025-01-21

**Authors:** Andrés Louloudes-Lázaro, Pablo Nogales-Altozano, José M. Rojas, Jeury Veloz, Ana B. Carlón, Piet A. Van Rijn, Verónica Martín, Ana Fernández-Sesma, Noemí Sevilla

**Affiliations:** 1 Centro de Investigación en Sanidad Animal, Instituto Nacional de Investigación y Tecnología Agraria y Alimentaria, Consejo Superior de Investigaciones Científicas (CISA-INIA-CSIC), Valdeolmos, Madrid, Spain; 2 Department of Microbiology, Icahn School of Medicine at Mount Sinai, New York, NY USA; 3 The Graduate School of Biomedical Sciences, Icahn School of Medicine at Mount Sinai, New York, NY USA; 4 Department of Virology, Wageningen Bioveterinary Research, Lelystad, The Netherlands; 5 Department of Biochemistry, Centre for Human Metabolomics, North-West University, Potchefstroom, South Africa

**Keywords:** cGAS, Interferon, RNA virus, Bluetongue, Autophagy, STING

## Abstract

Cyclic GMP-AMP synthase (cGAS) is a DNA sensing cellular receptor that induces IFN-I transcription in response to pathogen and host derived cytosolic DNA and can limit the replication of some RNA viruses. Some viruses have nonetheless evolved mechanisms to antagonize cGAS sensing. In this study, we evaluated the interaction between Bluetongue virus (BTV), the prototypical dsRNA virus of the Orbivirus genus and the *Sedoreoviridae* family, and cGAS. We found mitochondrial damage and DNA accumulation in the cytoplasm of infected cells. In addition, we show that BTV infection blocks DNA-induced IFN-I transcription and that virus infection prevents DNA sensing by inducing cGAS and STING degradation. We identify BTV-NS3 as the viral protein responsible for cGAS degradation, showing that NS3 physically interacts with cGAS and induces its degradation through an autophagy-dependent mechanism. Taken together, these findings identify for the first time a mechanism by which a dsRNA virus interferes with a DNA sensing pathway to evade the innate immune response.

**Supplementary Information:**

The online version contains supplementary material available at 10.1007/s00018-025-05580-5.

## Introduction

Activation of the innate immune response is the first mechanism for host defense to limit viral infection. One of the first steps involved in the activation of this response is the sensing of pathogen-associated molecular patterns (PAMPs) through cellular pattern recognition receptors (PRRs) [1–3]. One of the most common PAMPs sensed by PRRs during infection are viral nucleic acids and their intermediates produced during replication [4]. As a result of PRR activation, transduction signaling cascades are activated, resulting in diverse effects such as type-I interferon (IFN-I) induction, which leads to an antiviral state to combat infection [5]. Although it has been canonically understood that RNA sensing PRRs respond to RNA based pathogens and that DNA sensing PRRs respond to DNA based pathogens, the existence of a cross-talk between RNA and DNA cytoplasmic sensor pathways has now been demonstrated [6, 7]. Cyclic GMP-AMP synthase (cGAS) is a PRR that senses pathogen and host-derived DNA in the cytosol. cGAS activation leads to the production of 2’-3’ cyclic-guanosine monophosphate (GMP)-adenosine monophosphate (AMP) (cGAMP), a secondary messenger molecule that interacts with the stimulator of interferon genes (STING), inducing its dimerization and interaction with tank binding kinase 1 (TBK1) and the phosphorylation of the transcription factor interferon regulatory factor 3 (IRF3) [8], that translocates to the nucleus to induce type I IFN gene transcription [1]. cGAS can nonetheless also restrict viral RNA replication by sensing the accumulation of mitochondrial DNA (mtDNA) in the cytoplasm due to infection [9, 10]. Interestingly, some RNA viruses have evolved mechanisms that antagonize the cGAS pathway during infections to avoid mtDNA sensing by cGAS [11–13].

The *Sedoreoviridae* family includes viruses of great importance for human and animal health, such as rotavirus and orbivirus. These viruses possess a double-strand RNA (dsRNA) genome, which is typically sensed as a PAMP by PRRs such as cytosolic RNA sensors RIG-I, MDA-5 [14] and TLR3 [15], inducing an interferon response. Bluetongue virus (BTV) is the prototype of the orbivirus genus. It is an arthropod-borne virus transmitted by *Culicoides* spp. midges that mainly infects wild and domestic ruminants, causing a disease of compulsory declaration to the WOAH (World Organization for Animal Health) that leads to great economic losses for the livestock industry [16]. BTV possesses multiple serotypes that produce different degrees of disease severity in the ruminant host [17]. BTV-8, which is currently circulating in Europe, not only affects sheep but it can also produce disease in cattle, thus increasing its economic impact. The BTV genome consists of 10 segments of dsRNA encoding 7 structural proteins (VP1-7) and 5 non-structural proteins (NS1-5) [18, 19]. VP2 and VP5 proteins form the external layer of the virus and are involved in receptor binding and virus entry, VP3 and VP7 proteins provide structural integrity to the virions by forming the inner core, and VP1, VP4 and VP6 form the transcription complex and are involved in RNA transcription and viral replication [20]. Non-structural protein NS1 is involved in viral mRNA translation, NS2 is implicated in the formation of viral inclusion bodies, NS3 takes part in the release of the viral particles from the cell and acts also an IFN antagonist, NS4 is an IFN antagonist, and NS5 is likely to reduce host cell protein synthesis and support viral protein synthesis and replication [18, 20, 21].

Although BTV dsRNA sensing during infection induces an interferon response, the virus is still capable of replicating in the presence of IFN since it possesses diverse mechanisms for innate immune response evasion [22]. For instance, BTV-NS3 protein is an IFN antagonist that blocks IFN-I induction by interfering with RLR signaling pathway [23]. It also restricts interferon-stimulated gene transcription by promoting the degradation of STAT2, a cellular component of the IFN-I signaling pathway [24]. BTV-NS4 has also been described as an antagonist of IFN induction and IFN-I and-II signaling [21, 25].

In this work, we studied BTV as a prototypical dsRNA virus to examine if this virus could trigger DNA-sensing pathways during infection. BTV infection causes disruption of mitochondrial membrane and cytosolic DNA accumulation, which could potentially activate cGAS sensing of the infection. However, we show that BTV interferes with DNA-induced type-I interferon, and it degrades cGAS through an autophagy-dependent mechanism mediated by the NS3 protein. This study presents for the first time evidence that dsRNA viruses have also evolved developed mechanisms that prevent DNA sensing by disrupting the cGAS pathway.

## Materials and methods

### Cell lines and viruses

Human Embryonic Kidney (HEK)-293T cells (ATCC CRL-1573) were grown in Dulbecco’s Modified Essential Medium (DMEM) (Thermo Scientific) supplemented with 10% fetal bovine serum (FBS) (Sigma). HEK-293T cells stably transfected with a firefly luciferase reporter gene driven by the IFN-β promoter (293T–IFN-β–FFLuc), previously described in [26], were grown as stated for HEK-293T cells. BHK-21 (ATCC CRL-6281 C) and Vero (ATCC CCL-81) were grown in DMEM supplemented with 5% FBS. Peripheral blood monocyte (THP-1) cells (ATCC TIB-202) were grown in Roswell Park Memorial Institute (RPMI) medium (Thermo Scientific) supplemented with 10% FBS. Sheep thymus (ST) cells, an established cell line produced from primary thymus cells [27] were grown in DMEM supplemented with 10% FBS. All cells were kept at 37 °C in the presence of 5% CO_2_. Bluetongue virus serotype 8 (BTV-8, Belgium/06) was used in all the experiments. BTV stocks were prepared by infecting BHK-21 cells with a MOI of 0.1. Supernatants were collected at 48 h post-infection (hpi), and after freeze/thaw cycles and sonication, the supernatants were clarified and stored at -80ºC until use. Reverse genetic BTV-8 with a deficient NS3 encoding ORF (BTV-8 ΔNS3) and its control (rg BTV-8) were prepared as described by [28]. Modified Vaccinia Ankara virus (MVA) was kindly provided by Dr. Javier Ortego (CISA-INIA/CSIC).

### Virus inactivation

BTV-8 was inactivated with 1 mM binary ethyleneimine (BEI) at 37ºC for 48 h. Briefly, 1mM BEI was prepared by dissolving 2-bromoethylamine hydrobromide (BEA) in 0.7% sodium hydroxide (NaOH). The solution was incubated at 37ºC for 1 h to allow BEA to get converted to BEI, which was then sterilized using 0.2 μm filters. 3% BEI was added to virus suspension and the inactivation was carried out at 37ºC for 48 h with an intermediate change of flask at an 18-hour interval.

### Plasmids

Plasmids encoding BTV NS3 and NS4 open reading frame (ORF) were cloned into pIRES-cOFP-COOH-FLAG (Promega) expression vector. Plasmid encoding BTV NS3 protein (NS3-FLAG-pIRES-cOFP) was used as template to generate NS3 non-ubiquitinated mutant by substituting K amino acid residues 13 and 15 (NS3- K_13_,_15_), as described in [24]. pLenti-CMV-cGAS-HA plasmid, encoding human cGAS, was a gift from Jonathan Kagan (Addgene plasmid #130910; http://n2t.net/addgene:130910; RRID: Addgene_130910). pcDNA3.0-STING-FLAG plasmid, encoding human STING, was kindly provided by Dr. Subhash G Vasudevan (Duke-NUS Graduate Medical School, Singapore). pCAGGs-Denv-NS5-HA plasmid, encoding dengue virus NS5 protein was provided by Dr. García-Sastre (Icahn School of Medicine at Mount Sinai, New York, USA).

### Interferon production assays and quantitative PCR

ST cells were treated with mock or BEI inactivated BTV (iBTV) or infected with BTV-8 at a MOI of 5.0 (or its equivalent prior to inactivation for iBTV). Six hours after primary treatment, cells were either treated with mock, infected with MVA at an MOI of 2.0, or transfected with *E.coli* DNA (1 µg / 2.0 × 10^5^ cells) using TransIT^®^-LT1 (Mirus Bio). Twelve hours post-secondary treatment, nucleic acids were isolated using RNeasy Micro Kit (Qiagen) according to the manufacturer´s guidelines. Quantification of ovine *Ifnα* and *Isg15* transcripts was determined by normalizing gene expression to *β-actin* gene expression, and relative expression levels were calculated using the 2^−ΔΔCt^ method [29], where ∆Ct = Ct gene of interest (*Ifnα* or *Isg15*) – Ct housekeeping gene (*β-actin*) and ∆∆Ct = Sample ∆Ct average - Control group (mock) ∆Ct average). Quantitative real-time PCR (qPCR) and reverse transcription (RT)-qPCR were carried out to verify MVA and BTV infection respectively, using Luna Universal One-Step (New England Biolabs). For this purpose, cells were collected and pelleted to remove supernatant and washed twice with PBS (phosphate buffered saline) before nucleic acids isolation.

Transcription levels of *Ifnα*, *Isg15* and *β-actin* genes were evaluated by RT-qPCR using SYBR Green I Master Reagents (Roche). All PCRs were performed on a Light Cycler 480 System instrument (Roche). Primer sequences are available upon request.

### Interferon-ß reporter assay

HEK-293T cells stably expressing firefly luciferase under the control of an IFN-ß promotor (293T–IFN-β–FFLuc) were used to study the ability of BTV NS3 protein to inhibit the induction of the IFN-ß reporter. Briefly, 50,000 cells were transfected using TransIT^®^-LT1 (Mirus Bio) with 100ng total DNA composed of a mix of the following plasmids: pcDNA3.1-empty, pLenti-CMV-cGAS-HA, pcDNA3.1-STING, pIRES-BTV-NS3-FLAG or pIRES-BTV-K_13_,_15_/R-NS3-FLAG (K_13_,_15_/R-NS3 non-ubiquitinated mutant). Twenty-four hours post-transfection, IFN-ß promotor induction was measured using the neolite luminescence reporter gene assay system (PerkinElmer) as per the manufacturer’s protocol.

### Western blots

HEK-293T or THP-1 cells were mock treated or infected with BTV-8 (MOIs 0.1, 1.0 and 5.0) or transfected with our experimental control and control plasmids using TransIT^®^-LT1 (Mirus Bio), following the manufacturer´s protocol. Twenty-four hours later, cells were washed with PBS and lysed on ice for 30 min with RIPA lysis buffer (Sigma) supplemented with complete protease inhibitor (Roche). Lysates were centrifuged for 15 min at 20,000 xg at 4ºC to remove cell debris. Cell lysates were re-suspended, in 2X Laemmli sample buffer (Bio-Rad) supplemented with 2-mercaptoethanol and boiled at 100 °C for 10 min. The samples were loaded in polyacrylamide–SDS gels and the proteins were electrophoretically separated. Proteins were then transferred to PVDF membranes. Blots were blocked with tris-buffered saline (TBS) with 0,05% Tween 20 (Thermo Scientific) and 5% milk for one hour at room temperature (RT). Blots where then incubated with the following primary antibodies: anti-cGAS (D1D3G), anti-STING (D2P2F), anti-ATG7 (D12B11), anti-LC3A/B (D3U4C) (all from Cell Signaling Technology), anti-FLAG (F7425), anti-ß-actin (A2228) and anti-haemagglutinin (HA) (H3663) (all from Sigma Aldrich). Secondary HRP-conjugated anti-mouse or anti-rabbit IgG (GE Healthcare) antibodies were used, and protein bands were visualized by chemiluminescence (ECL Plus, Thermo Scientific). Immunoblot image acquisition was performed on a ChemiDoc Imaging System (Biorad). Densitometry analysis of cGAS, STING and β-actin expression was performed using ImageJ software.

### Proteasome and autophagy pathway inhibition

HEK-293T cells were transfected with plasmids encoding cGAS and BTV-NS3 or an empty vector. Twenty-four hours post-transfection, cells were treated with proteasome inhibitors MG132 (0.2, 2 or 20 µM) and lactacystin (20 µM), autophagosome formation inhibitor 3-MA (5 mM) (all from Sigma), or autophagosome maturation inhibitor, bafilomycin A1 (BAF-A1, 1, 10 or 100 nM) (InvivoGen). Six hours post-treatment, cells were lysed, and proteins were analyzed via Western blot. For *ATG7* gene silencing, HEK-293T cells were transfected with 100 nM of *ATG7* siRNA or control siRNA (Cell Signaling Technology) for 48 h and transfected with plasmids encoding cGAS and BTV-NS3 for an additional 24 h. All transfections were carried out using jetPRIME^®^ transfection reagent (Polyplus). Cell lysates were collected and analyzed by SDS-PAGE and immunoblotting.

### cGAS inhibition assay

THP-1 cells were mock treated of infected with BTV-8 (MOI 0.1) and 1 h after infection cells were treated with cGAS inhibitor G140 (20 µM) (InvivoGen). At 16 h post-infection, cells were harvested for determination of viral replication by plaque assay and qPCR.

### Immunoprecipitation assays

HEK-293T cells were co-transfected with plasmids encoding HA-tagged cGAS and FLAG-tagged BTV-NS3. Twenty-four hours post-transfection, cell lysates were collected as described in the Western blot section. A portion of the lysates was separated and kept as whole cell extract (WCE), while the other portion was used to perform the immunoprecipitation (IP) assay. Lysates were incubated with anti-HA or anti-FLAG affinity gel antibody beads (Sigma) overnight at 4ºC under rolling agitation. After incubation, the affinity gel samples were washed four times for 10 min in lysis buffer. The affinity gel samples were then re-suspended in 2X Laemmli buffer with 2-mercaptoethanol and boiled at 100ºC for 10 min. SDS–PAGE followed by immunoblot analysis was performed on the IP and WCE.

### Immunofluorescence

Vero cells were seeded on coverslips (Thermo Scientific) in 12-well plates and then mock treated or infected with BTV-8 at an MOI of 5.0. Twenty-four hpi, cells were washed three times with PBS before fixing and permeabilizing with 100% ice cold Methanol (Thermo Scientific) for 10 min at -20ºC. Nonspecific binding sites were blocked with Dako antibody diluent (Agilent) for 1 h at RT. Cells were incubated with primary antibodies overnight at 4ºC, after which they were washed three times with PBS and incubated for 1 h at RT with secondary antibodies. After washing, nuclei were stained with 10 µg/ml 4′,6-diamidino-2-phenylindole (DAPI) (Sigma) and coverslips were mounted using Prolong Gold antifade reagent (Invitrogen). The following antibodies were used: primary antibodies anti-VP2-BTV8 rabbit serum produced in our laboratory [30], anti-ssDNA mouse (MAB3034: EMD Millipore), anti-TOM20 mouse (SC17764, Santa Cruz), and secondary antibodies anti-mouse IgG conjugated to Alexa Fluor 647 and anti-rabbit IgG conjugated to Alexa Fluor 488 (Invitrogen).

### Confocal microscopy image acquisition

Images were captured using a LSM 880 confocal microscope (Zeiss) fitted with a Plan Apochromatic ×63/1.4 or ×40/1.4 oil objective. Images were collected at a resolution of 1,024 × 1,024 pixels. Image processing was carried out using Fiji/Image J software.

### Mitochondrial membrane potential assay

JC-1 - Mitochondrial Membrane Potential Assay Kit (Abcam) was used to evaluate the ability of BTV to modify mitochondrial membrane potential. Tetraethylbenzimidazolylcarbocyanine iodide (JC-1) is a cationic dye that accumulates in energized mitochondria. At low concentrations (due to low mitochondrial membrane potential), JC-1 is predominantly a monomer that yields green fluorescence with emission of 530 ± 15 nm.

At high concentrations (due to high mitochondrial membrane potential), the dye aggregates yielding a red to orange colored emission (590 ± 17.5 nm). Therefore, a decrease in the aggregate fluorescent count is indicative of depolarization whereas an increase is indicative of hyperpolarization. Following the manufacturer´s guidelines, ST cells were seeded in 24-well plates and mock-treated or infected with BTV at MOIs of 0.1, 1.0 and 5.0. For iBTV treatment, cells were incubated with the equivalent to a MOI of 5 prior to inactivation. Twenty-four hpi, cells were treated with 25 µM tetraethylbenzimidazolylcarbocyanine iodide (JC-1) for 10 min at 37ºC, after which fluorescence was analyzed by flow cytometry. As a depolarization control, cells were treated with 100 µM carbonyl cyanide 4-(trifluoromethoxy) phenylhydrazone (FCCP) for 1 h at 37ºC prior to JC-1 addition. Samples were acquired on a BD FACSCelestaSorp flow cytometer and analyzed using the FlowJo software (Tree Star Inc, USA).

### Intracellular BTV-VP7 staining for detection of cell infection

Intracellular staining for BTV VP7 protein was carried out to confirm BTV infection of ST or HEK-293T cells in mitochondrial membrane potential assay and cGAS degradation assay, respectively. Twenty-four hours post-infection, cells were fixed and permeabilized with BD Cytofix/Cytoperm™ (BD Biosciences) for 20 min on ice. After two washes with 1×BD Perm/Wash™ buffer (BD Biosciences), cells were stained with anti-BTV-VP7-FITC-conjugate mouse antibody (VMRD) for 30 min on ice. After two more washes with PBS, cells were analyzed by flow cytometry.

### Statistical analysis

Statistical analysis was carried out using Prism 8.0 software (GraphPad Software Inc). Data were analyzed with one-way ANOVA with Tukey´s multiple comparisons test. P-values were considered statistically significant when *p* < 0.05.

## Results

### Bluetongue virus causes mitochondrial membrane depolarization and induces cytosolic DNA accumulation

**Fig. 1:**
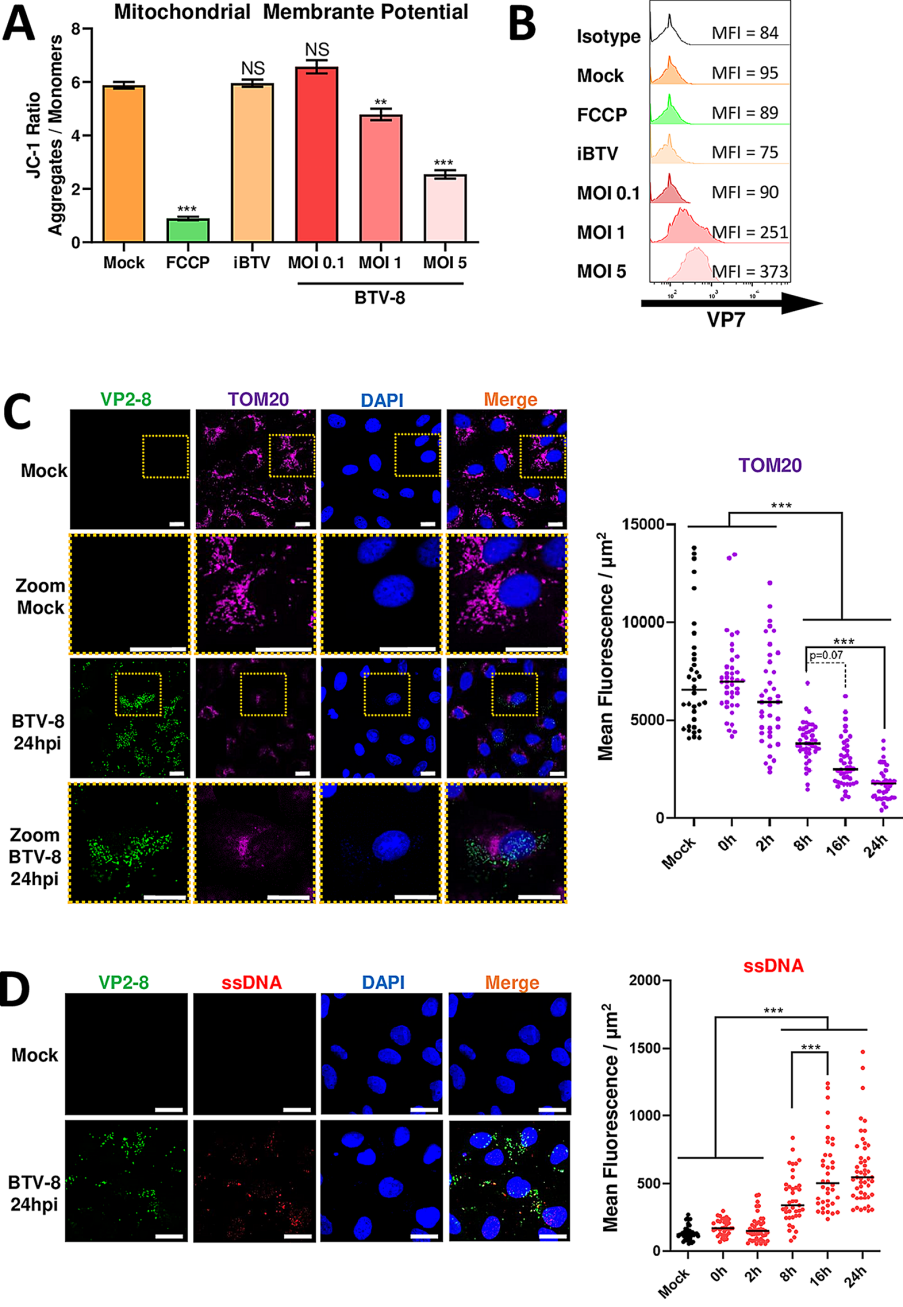
BTV infection disrupts mitochondrial membrane potential and induces cytosolic DNA accumulation. (**A**). Mitochondrial membrane potential assay. ST cells were mock-treated, infected at different MOIs of BTV-8 (0.1, 1 and 5) or inactivated BTV (iBTV). Twenty-four hours post-infection, cells were treated with JC-1, a cationic dye that aggregates in energized mitochondria yielding a red to orange colored emission (590 ± 17.5 nm) whereas when mitochondrial membrane is compromised, it predominantly remains in the cytosol as a monomer that emits green fluorescence (530 ± 15 nm). (FCCP) treatment (100µM for 1 h) was used as a depolarization control. Fluorescence was analyzed by flow cytometry and data of three independent experiments (*n* = 3) are represented as the mean ± SD of JC-1 Ratio of aggregates/monomers. (**B**). A portion of the cells from A were fixed and stained with anti-BTV-VP7 antibody and then analyzed by flow cytometry in order to verify BTV infection and geometric mean fluorescence intensity (MFI) of VP7 + cells is represented. (**C**-**D**). Analysis of mitochondrial distribution and morphology and detection of cytoplasmic DNA by immunofluorescence. Vero cells were mock-treated or infected with BTV-8 (MOI 5) and 0, 2, 8, 16–24 h post-infection (hpi) cells were fixed and stained for BTV-8 VP2, and (**C**) mitochondrial marker TOM20, or (**D**) ssDNA. Nuclei were stained with DAPI. Scale bar = 20 μm. TOM20 or ssDNA marker fluorescence at the different times post-infection was measured by quantifying the mean fluorescence and the area (µm^2^) of at least 40 cells (*n* ≥ 40) for each condition. Statistical analysis was carried out with one-way ANOVA with Tukey´s multiple comparisons test, comparing each condition to mock condition. Statistical significance is represented as: NS = not significant; ** = *p* < 0.01; *** = *p* < 0.001

cGAS has been described as a DNA sensor that induces type-I interferon production in response to pathogen and host-derived DNA. Cell stress during viral infection can lead to release of DNA into the cytoplasm, activating cGAS and inducing an interferon response to combat the infection [11, 13]. To study whether BTV infection could result in mitochondrial membrane destabilization and cytosolic DNA accumulation that would induce cGAS activation, we infected ovine ST cells with BTV-8 at MOIs of 0.1, 1 and 5 and assessed mitochondrial membrane potential with JC-1. When this cationic dye is transported into energized mitochondria, it forms aggregates that yield an orange-colored emission (590 ± 17.5 nm), whereas when mitochondrial membrane is compromised, it predominantly remains in the cytosol as a monomer that yields a green fluorescence emission (530 ± 15 nm) [31]. We therefore compared the JC-1 ratio of aggregates / monomers by flow cytometry to study the mitochondrial membrane potential of BTV-infected cells (Fig. 1A). We observed that mock treated cells showed the highest JC-1 aggregate / monomer ratio, while depolarized FCCP treated cells showed the lowest. In BTV-infected cells, the JC-1 aggregate / monomer ratio decreased as MOI increased, which indicates that BTV infection compromised the mitochondrial membrane. Furthermore, treatment with BEI-inactivated BTV (iBTV) did not alter the mitochondrial membrane potential, indicating that viral replication is necessary to induce mitochondrial membrane destabilization. BTV infection in these experiments was confirmed by flow cytometry with anti-BTV-VP7 antibody (Fig. 1B).

In order to visualize alterations in mitochondria during BTV infection, we carried out an immunofluorescence analysis in which Vero cells were mock treated or infected with BTV-8 at MOI of 5, fixed at 0, 2, 8, 16 and 24 hpi and then probed with anti-BTV-VP2 rabbit serum and anti-TOM20 mitochondrial marker (Fig. 1C). We observed alterations in mitochondrial morphology in BTV-infected cells displaying more diffused structures than in mock treated cells, which became more evident at 24 hpi (Fig. 1C). Quantification of mitochondrial marker TOM20 started to show a decrease in mean fluorescence at 8 hpi when compared to mock treated cells, which continued to gradually decrease until 24 hpi (Fig. 1C). In addition, to evaluate whether BTV infection could cause DNA leakage into the cytosol and potentially induce cGAS activation, we carried out immunofluorescence analysis of BTV-infected or mock-infected cells stained with anti-ssDNA antibody, which was also quantified by measuring mean fluorescence at 0, 2, 8, 16 and 24 hpi (Fig. 1D). A positive control for ssDNA leakage and staining was obtained with irradiated cells (Supplementary Fig. 1). Immunofluorescence data indicate that BTV infection starts to induce DNA accumulation in the cytosol of infected cells at 8 hpi, which significantly increases until 24 hpi (Fig. 1D). This could possibly occur as a consequence of mitochondrial membrane depolarization (Fig. 1).

### BTV reduces DNA-induced type-I interferon production

**Fig. 2:**
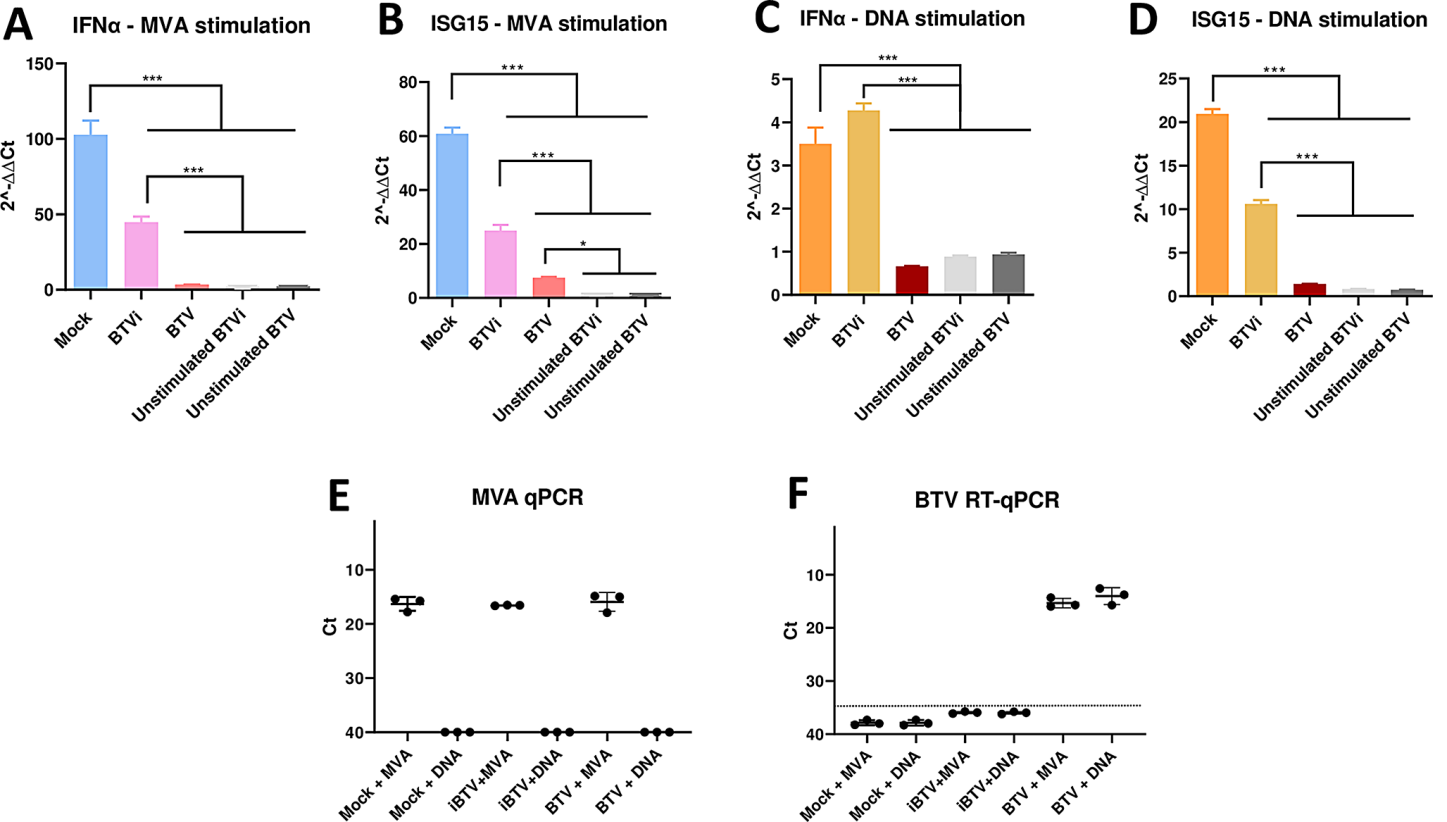
Bluetongue virus interferes with DNA induced type-I interferon production. Sheep thymus cells (ST) were treated with mock, inactivated BTV (iBTV) or infected with BTV-8 (MOI 5). 6 h post-infection, cells were infected with (**A**-**B**) MVA (MOI 2) or (**C**-**D**) transfected with *E.coli* DNA (1 µg / 2 × 10^5^ cells) for 12 h. (A-D). Quantification of *Ifnα* and *Isg15* transcripts by qPCR. Cell lysates were collected for RNA isolation and RT-qPCR quantification of *Ifnα* and *Isg15* transcripts. Data of three independent experiments (*n* = 3) are represented as relative expression using the 2^−∆∆Ct^ method. Statistical analysis was carried out with one-way ANOVA with Tukey´s multiple comparisons test. Statistical significance is represented as: NS = not significant; ** = *p* < 0.01; *** = *p* < 0.001. (**E**-**F**). Quantification of MVA DNA, by qPCR by amplifying a 281pb fragment from MVA genome, and BTV RNA by RT-qPCR, by amplifying a 165pb fragment of the segment 5 of BTV. Cell lysates were collected after removing supernatant and washing cell pellet with PBS. Nucleic acids were isolated from the lysates to quantify (**E**) MVA genomic DNA by qPCR and (**F**) BTV genomic RNA by RT-qPCR. Data are represented by means ± SD. Dotted line in panel F indicates RT-qPCR detection limit for BTV RNA

Previous studies have shown that BTV possesses multiple mechanisms that block IFN-I transcription to evade the immune response [21, 23, 24]. However, because BTV is an RNA virus, these studies have focused on evaluating the interaction of this virus with cellular components of RNA-sensing pathways. Our data show that BTV infection results in cytosolic DNA accumulation. For this reason, we evaluated whether BTV is capable of inhibiting IFN-I induction through DNA-sensing pathways. Accordingly, ST cells were infected with BTV-8 at a MOI of 5, mock-treated, or treated with inactivated BTV (iBTV). At six hpi, cells were infected with MVA or transfected with *E. coli* DNA to activate DNA-sensing pathways like the cGAS cascade [32]. Sixteen hours later, we evaluated by RT-qPCR transcription levels of *Ifnα* and *Isg15* genes (Fig. 2A-D). Results showed that BTV infection produces a drastic decrease of *Ifnα* and *Isg15* transcription levels induced by MVA infection and DNA transfection. Transcription levels of *Ifnα* and *Isg15* genes at earlier infection timepoints (2 h post-BTV infection) did not show significant differences with later timepoints (Supplementary Fig. 2), more likely due to the methodology employed, since measurement of these IFN-related genes requires a 12 h stimulation with MVA, i.e. gene expression was measured after a total of 14 h of BTV infection. In previous work using luciferase assays, we and others found that BTV infection fully blocks IFN promotor activity after 16 h of infection [23, 24]. A small decrease of *Ifnα* and *Isg15* transcription was also observed in iBTV treated cells, which could be due to structural components of the virus that have been described to interfere with interferon induction [23]. MVA and BTV infection was confirmed in these samples by qPCR and RT-qPCR respectively (Fig. 2E and F). Importantly, BTV replication in ovine cells led to the highest suppression of *Ifnα* and *Isg15* transcription, in comparison with iBTV, which shows that the infection suppresses IFN induction mediated by DNA sensing.

### BTV induces cGAS degradation during infection through the NS3 protein

**Fig. 3:**
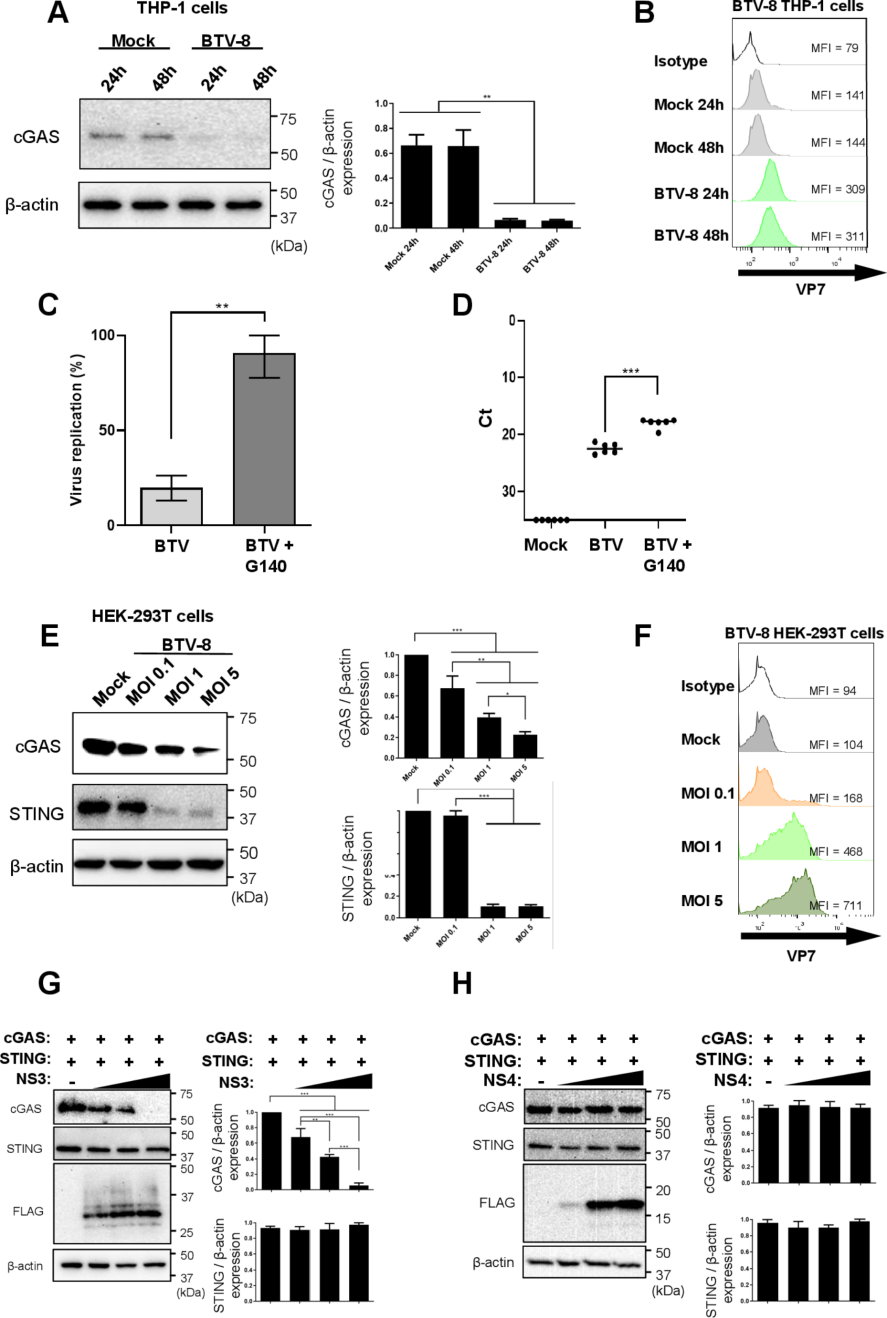
Bluetongue virus interferes with cGAS pathway by degrading cGAS through the NS3 protein. (**A**). THP-1 cells were infected with BTV-8 (MOI 1). 24- and 48-hours post-infection, endogenous cGAS expression was analyzed by SDS-PAGE and immunoblotting with cGAS and β-actin antibodies (**A**). (**C**-**D**). THP-1 cells were infected with BTV-8 (MOI 0.1) and one hour later, medium was replaced with fresh medium containing cGAS inhibitor G140 (20 µM) or mock-treated. 16 h post-treatment, cells were collected to determine viral titers by analyzing percentage of viral replication by plaque assay (**C**) or qPCR (**D**). Data in (**C**) and (**D**) represent the mean ± SD of at least three independent experiments performed in duplicates(**E**). HEK-293T cells were transfected with cGAS and STING encoding plasmids for 16 h and then treated with mock or infected with BTV-8 (MOI 0.1, 1 or 5). 24 h post-infection, cell lysates were collected and protein expression was visualized via SDS-PAGE and immunoblotting with cGAS, STING and β-actin antibodies. (**B**, **F**). A portion of the cells from A and E were fixed and stained with anti-BTV-VP7 antibody and then analyzed by flow cytometry to verify BTV infection. Geometric mean fluorescence intensity (MFI) of VP7 + cells is represented. (**G**, **H**). HEK-293T cells were transfected with plasmids encoding cGAS, STING and increasing concentrations of plasmids encoding BTV NS3-FLAG (**G**) or NS4-FLAG (**H**) (250, 500 and 1000ng). 24 h post-transfection cell lysates were analyzed by SDS-PAGE and immunoblotting with cGAS, STING, FLAG-tag and β-actin antibodies. Densitometry in analysis of cGAS and STING relative to β-actin is expressed in A, E, G and H as a percentage using ImageJ software. Representative images of at least 3 individual experiments. Statistical analysis was carried out with one-way ANOVA with Tukey´s multiple comparisons test. Statistical significance is represented as: * = *p* < 0.05; ** = *p* < 0.01; *** = *p* < 0.001

To determine whether BTV infection affected the cGAS-STING pathway to decrease the aforementioned DNA-induced IFN-I induction, we used THP-1 cells that express cGAS at endogenous levels detectable by specific antibody staining. To this end, THP-1 cells were infected with BTV at MOI 1. Western blot analyses of cell lysates showed endogenous cGAS in mock treated cells, and cGAS degradation at 24-and 48-hpi in BTV-infected THP-1 cells (Fig. 3A). BTV infection of THP-1 cells was confirmed by flow cytometry with an anti-VP7 antibody staining (Fig. 3B). These data indicate that BTV can degrade endogenous cGAS during infection.

To evaluate whether this degradation has a relevant biological role during infection or is a by-product of the infective process, we inhibited cGAS activity with the G140 inhibitor and measured viral replication. This inhibitor binds to cGAS active site and interrupts its enzymatic activity [33]. Consequently, THP-1 cells were infected with BTV-8 at MOI 0.1 and at 1 hpi, cells were treated with vehicle (DMSO) or with the G140 inhibitor. 16 h later, cells were collected to determine viral replication by plaque assay (Fig. 3C) and qPCR (Fig. 3D). We detected an 80% increase in plaque formation and increased viral RNA amounts when cells were treated with G140, showing that cGAS inhibition lead to higher BTV replication. These data therefore indicate that cGAS plays a restrictive role in BTV replication during infection, which could therefore favor the evolution of viral mechanisms, such as cGAS degradation, to by-pass this PRR pathway.

We next aimed to identify those viral proteins that might be implicated in cGAS degradation. To carry out these experiments we used HEK-293T cells due to their high transfection efficiency. Because endogenous cGAS was not detected in HEK-293T cells, we needed to transfect these cells with plasmids encoding cGAS or STING. First, we determined whether HEK-293T showed cGAS degradation after BTV infection, as we had shown with THP-1 cells. To this end, transfected HEK-293T cells with plasmid encoding cGAS or STING and mock-treated or infected them with BTV-8 at MOIs 0.1, 1 and 5. Twenty-four hpi, cell lysates were collected and protein expression was analyzed by SDS-PAGE followed by Western blotting. Densitometry analysis was performed to compare cGAS and STING expression to β-actin expression (Fig. 3E). BTV infection was confirmed by flow cytometry with an anti-BTV-VP7 antibody staining (Fig. 3F). We observed a decrease in the expression of both cGAS and STING when BTV MOI increased. Whilst we detected a 5-fold decrease in cGAS expression at a MOI of 5, we also observed a greater decrease of 10-fold in STING expression at a MOI of 1, which was maintained at a MOI of 5. This result indicates that the amount of both cGAS and STING is reduced by BTV infection (Fig. 3E).

We proceeded to identify the viral proteins involved in this process with NS3 and NS4 proteins as promising candidates, since these have been described as the two main BTV proteins acting as IFN-I antagonist [21, 23, 25, 34]. Our laboratory has also described that NS3 induces degradation of other components of the IFN-I induction pathway [24]. We studied whether these two viral proteins enable cGAS and STING degradation, thereby preventing DNA sensing during the infection. For this purpose, we transfected cells with plasmids encoding cGAS, STING and BTV-NS3 or BTV-NS4. Cell lysates were analyzed by SDS-PAGE and Western blotting, and densitometry analysis was performed to compare cGAS to β-actin expression (Fig. 3G and H). We observed a significant decrease in cGAS expression correlating with the increase of NS3 concentration. In contrast, cGAS expression was not altered in the presence of NS4, another non-structural protein, which is also an IFN-I antagonist [21]. These results indicate that BTV-NS3 protein is responsible for cGAS degradation in a dose-dependent manner. We did not detect any significant alterations in STING expression with NS3 or NS4, and therefore we decided to focus on the effects of cGAS expression in the presence of BTV-NS3.

**Fig. 4:**
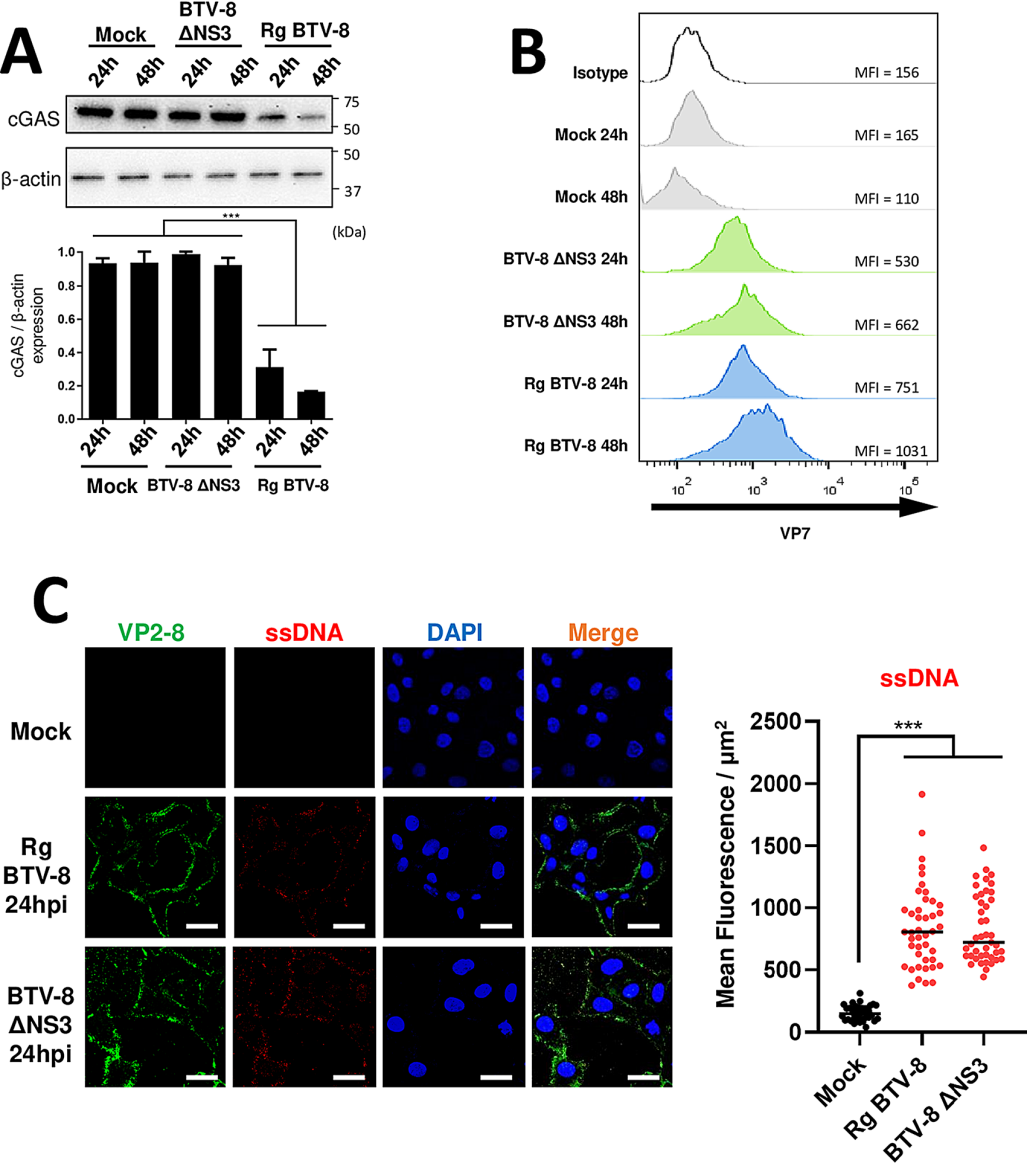
NS3-defective BTV does not degrade cGAS but induces cytosolic DNA accumulation. (**A**, **B**). HEK-293T cells were transfected with a plasmid encoding cGAS and 16 h later mock-infected or infected with reverse genetic BTV-8 with a defective NS3 encoding segment (BTV‐8 ΔNS3) or with reverse genetic BTV‐8 control (rgBTV-8). 24 and 48 h post-infection, a portion of the cells were lysed in order to analyze protein expression by SDS-PAGE followed by immunoblotting with cGAS, and β-actin antibodies (**A**), while the other portion of the cells were fixed and stained with anti-BTV-VP7 antibody and analyzed by flow cytometry to confirm BTV infection (**B**). Geometric mean fluorescence intensity (MFI) of VP7 + cells is represented in B. Densitometry in analysis of cGAS relative to β-actin is expressed in A using ImageJ software. Representative images of at least 3 individual experiments. Statistical analysis was carried out with one-way ANOVA with Tukey´s multiple comparisons test. Statistical significance is represented as: * = *p* < 0.05; ** = *p* < 0.01; *** = *p* < 0.001. (**C**). Detection of cytoplasmic DNA accumulation during NS3-defective BTV infection by inmunofluorescence. Vero cells were mock-treated or infected with reverse genetic BTV-8 with a defective NS3 encoding segment (BTV‐8 ΔNS3) or with reverse genetic BTV‐8 control (rgBTV-8) (MOI5), and 24 h post-infection cells were fixed and stained for BTV-8 VP2 and ssDNA marker. Nuclei were stained with DAPI. Scale bar = 20 μm. These data indicate that cytosolic DNA accumulation is independent of NS3 expression by BTV

To further confirm the role of NS3 in cGAS degradation during virus infection, HEK-293T cells were transfected with a plasmid encoding cGAS and infected with BTV-8 ΔNS3, a reverse genetic BTV-8 with a defective NS3 encoding segment, or its reverse genetic BTV-8 control (Rg BTV-8) and mock-infected. Protein expression was visualized by immunoblotting and cGAS expression was compared to β-actin expression by densitometry analysis (Fig. 4A). BTV-8 ΔNS3 or Rg BTV-8 infection was confirmed by flow cytometry by staining a fraction of the cells with anti-BTV-VP7 antibody (Fig. 4B). Results clearly showed a decrease in cGAS expression as Rg BTV-8 concentration increased but remained unaltered in BTV-8 ΔNS3-infected cells, confirming the critical role of NS3 protein in the degradation of cGAS during BTV infection. We also found that BTV-8 ΔNS3 infection still produced DNA accumulation in the cytoplasm of infected (Fig. 4C), thus excluding NS3 as the sole viral factor responsible for this accumulation.

### BTV NS3 interacts with cGAS and induces its degradation through an autophagy-dependent mechanism

**Fig. 5:**
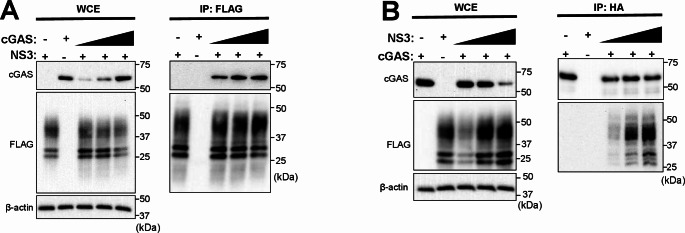
BTV NS3 protein interacts with cGAS. BTV-NS3 and cGAS co-immunoprecipitation assay. HEK-293T cells were co-transfected with (**A**) a plasmid encoding NS3-FLAG and increasing concentrations of a plasmid encoding cGAS-HA or with (**B**) cGAS-HA plasmid and increasing concentrations of NS3-FLAG plasmid. 24 h post-transfection, a fraction of the cell lysate was collected as whole cell extract (WCE) and the other fraction was incubated with (**A**) anti-FLAG or (**B**) anti‐HA affinity gel antibody beads to perform immunoprecipitation (IP) assays. Protein interactions were visualized via SDS-PAGE followed by immunoblotting

**Fig. 6:**
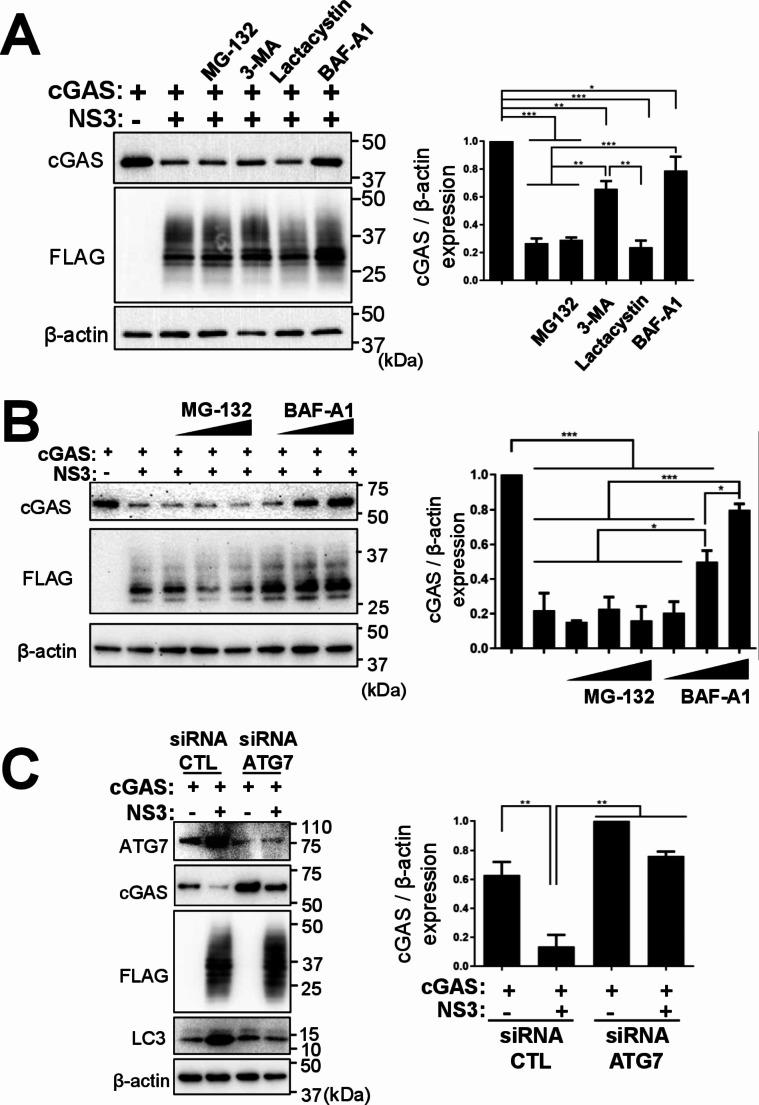
BTV NS3 protein induces cGAS autophagic degradation. (**A** – **B**). HEK-293T cells were transfected with plasmids encoding cGAS and BTV-NS3 an empty vector as control. 24 h post-transfection, cells were mock-treated or treated with proteasome inhibitors MG-132 (10 µM) and lactacystin (20 µM) or autophagy inhibitors 3-MA (5 mM) and BAF-A1 (100 nM) (**A**) or with increasing concentrations of proteasome inhibitor MG-132 (0.2, 2 or 20 µM) or increasing concentrations of autophagy inhibitor BAF-A1 (1, 10 or 100 nM) (**B**). 6 h post-treatment, cell lysates were analyzed by SDS-PAGE and immunoblotting with cGAS, FLAG-tag and β-actin antibodies. (**C**). Autophagy inhibition assay. HEK-293T cells were transfected with control (CTL) siRNA or with ATG7 siRNA for 48 h and transfected with plasmids encoding cGAS or BTV-NS3-FLAG for 24 h. Cell lysates were analyzed by SDS-PAGE and immunoblotting using indicated antibodies. Densitometry in analysis of cGAS or STAT2 relative to β-actin is expressed in (**A**-**D**) as a percentage using ImageJ software. Representative images of at least 3 individual experiments. Statistical analysis was carried out with one-way ANOVA with Tukey´s multiple comparisons test. Statistical significance is represented as: * = *p* < 0.05; ** = *p* < 0.01; *** = *p* < 0.001

Considering previous studies from our laboratory in which NS3 was shown to bind to STAT2, a cellular component of the IFN signaling pathway, and induce its autophagic degradation [24], we evaluated if NS3 could produce similar interactions with cGAS. Firstly, we performed an immunoprecipitation (IP) assay of cells transfected with HA-tagged cGAS and FLAG-tagged NS3 (Fig. 5A and B). Western blot analysis showed that cGAS co-immunoprecipitated with NS3 when IP assay was performed with anti-FLAG affinity antibody beads (Fig. 5A) and that NS3 co-immunoprecipitated with cGAS when IP assay was performed with anti-HA affinity antibody beads (Fig. 5B). These results show that NS3 interacts with cGAS. To assess which cellular pathways are implicated in NS3-mediated degradation of cGAS, cells transfected with plasmids encoding cGAS and BTV-NS3 were treated with proteasome and autophagy inhibitors. Results revealed that treatment with proteasome inhibitors MG-132 and lactacystin did not prevent NS3-mediated cGAS degradation. However, treatment with autophagosome formation inhibitor 3-MA and autophagic maturation vacuole inhibitor bafilomycin A1 (BAF-A1) led to cGAS recovery in the presence of NS3 (Fig. 6A). In addition, when cells were treated with increasing concentrations of BAF-A1 cGAS degradation in the presence of BTV-NS3 was reduced in a dose-dependent manner, which did not occur in the presence of increasing concentrations of MG-132 (Fig. 6B). cGAS NS3-mediated degradation was therefore blocked by treatment with autophagy inhibitors but not by proteasome inhibitors. Using DenV-NS5 transfection in HEK-293 cells, a viral protein that mediates STAT2 degradation through the proteasome [35], we confirmed that the proteasome inhibitors were functional at the dose used in our studies (Supplementary Fig. 3). To further study the involvement of autophagic pathways in NS3-mediated cGAS degradation, knockdown of autophagy related protein 7 (ATG7), a critical component in the formation of phagophores [36], was carried out in cGAS and NS3 expressing cells by siRNA gene silencing. Western blot analysis revealed that when ATG7 protein expression was inhibited by siRNA silencing cGAS expression was restored in presence of BTV-NS3 protein (Fig. 6D), supporting the previous results. Furthermore, we observed that ATG7 and the microtubule-associated protein 1 A/1B-light chain 3 (LC3) lower molecular weight band accumulated in NS3-transfected cells when co-transfected with control siRNA, which could indicate that the viral protein is in fact an autophagy inducer. Taken together, these results indicate that NS3 induces cGAS degradation through an autophagy-dependent mechanism.

### BTV-NS3 protein requires ubiquitination to efficiently degrade cGAS and interfere with IFN-I production

**Fig. 7:**
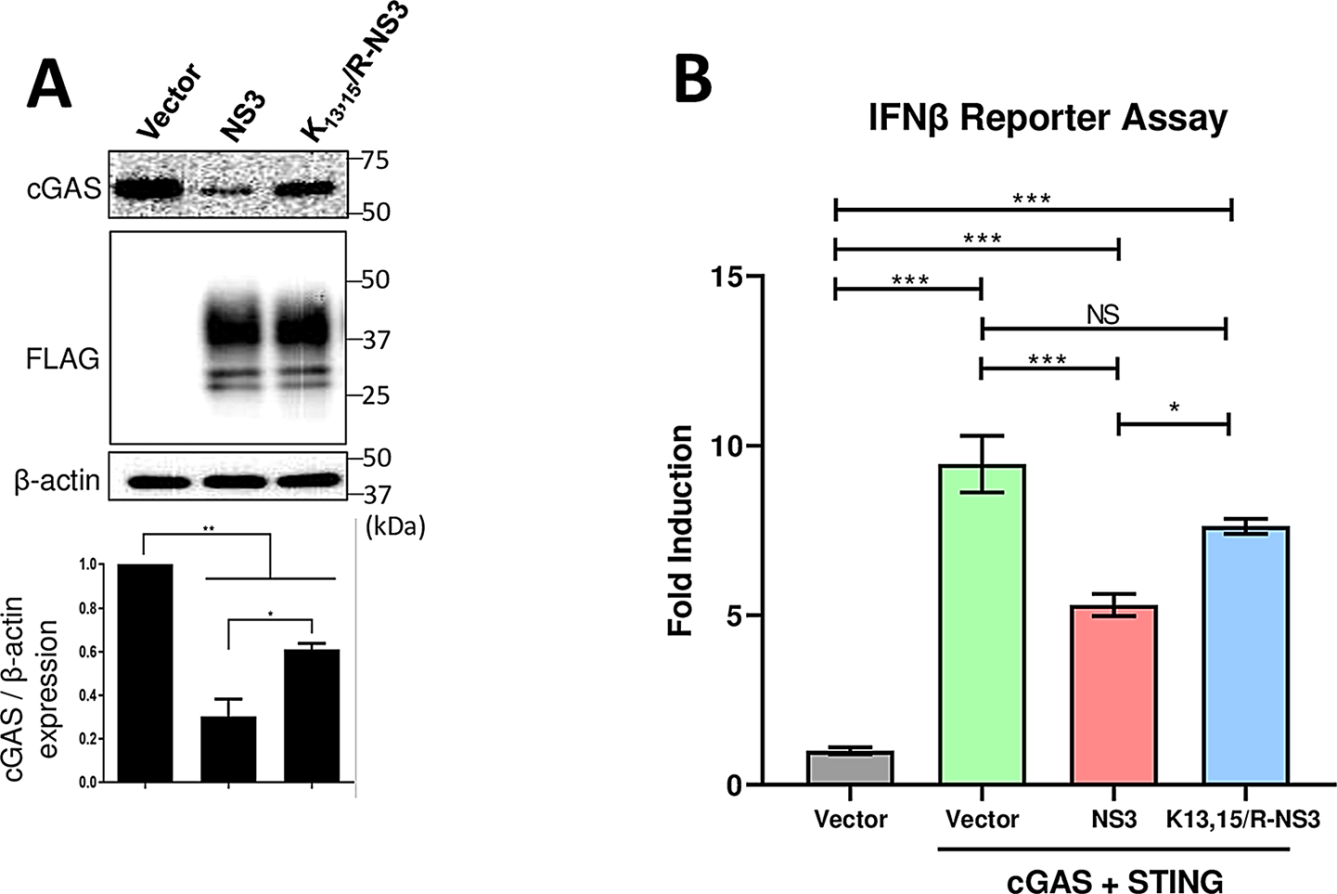
BTV NS3 ubiquitination is required for efficient cGAS degradation and inhibition of IFN-I transcription. (**A**). NS3 non-ubiquitinated mutant is less efficient in cGAS degradation. HEK-293T cells were transfected with plasmids encoding cGAS and BTV wild type NS3 or non-ubiquitinated NS3 mutant (K13,15/R-NS3). 24 h post-transfection, protein expression was visualized by SDS-PAGE and immunoblotting with cGAS, FLAG-tag and β-actin antibodies. Densitometry in analysis of cGAS relative to β-actin is expressed as a percentage using ImageJ software. (**B**). Evaluation of type-I interferon production of cells expressing BTV NS3 protein. 293T–IFN-β–FFLuc cells were transfected with an empty vector or plasmids encoding cGAS and STING and an empty vector, wild type NS3, or non-ubiquitinated NS3 mutant (K13,15/R-NS3). 24 h post-transfection, cell lysates were collected for luminescence quantification. Data representative of three independent experiments (*n* = 3) are represented means ± SD of fold induction over the empty vector condition. Statistical analysis was carried out with one-way ANOVA with Tukey´s multiple comparisons test. Statistical significance is represented as: NS = not significant; * = *p* < 0.05; *** = *p* < 0.001

Avia, Rojas (24) described that BTV-NS3 is ubiquitinated on lysine residues 13 and 15, and that protein ubiquitination is required for E3 ligase recruitment in order to induce autophagic degradation of cellular components of the IFN-I signaling pathway. Therefore, we studied whether NS3 ubiquitination is relevant in the degradation of cGAS. Hence, cells were transfected with plasmids encoding cGAS and wild-type NS3 or a non-ubiquitinated NS3 mutant (K_13_,_15_/R-NS3, with lysine residues 13 and 15 substituted for arginine residues). Western blot and densitometry analysis (Fig. 7A) revealed that cGAS was degraded to a lesser extent in the presence of K_13_,_15_/R-NS3 when compared to wildtype NS3, indicating that NS3 ubiquitination is required for efficient degradation of cGAS. Additionally, the importance of NS3 ubiquitination in inhibition of IFN-I induction through the cGAS pathway was evaluated. To this end, we used HEK-293T cells stably transfected with a firefly luciferase reporter gene driven by the IFN-β promoter (293T–IFN-β–FFLuc). These cells were transfected with equal amounts of plasmids encoding cGAS and STING (to express the main components of the cGAS pathway in order to induce interferon production) and an empty vector as a positive control or wildtype NS3 or K_13_,_15_/R-NS3; or an empty vector as a negative control. Luciferase activity was measured to study IFN-β production (Fig. 7B). The reporter assay showed that although IFN-β production decreased in the presence of K_13_,_15_/R-NS3, wildtype NS3 interfered more efficiently with IFN-β induction. This indicates that NS3 ubiquitination is essential for effective cGAS degradation and IFN-I transcription antagonization.

## Discussion

cGAS has been characterized as a DNA cytosolic sensor that was thought to exclusively respond to DNA virus infection [37, 38]. However, such activation has also been described during RNA virus infection due to cellular DNA accumulation in the cytoplasm [9, 39, 40]. Since cGAS activation can limit viral RNA replication, some RNA viruses can evade PAMP sensing by targeting cGAS [11, 13, 41, 42]. BTV, which a dsRNA virus, can be sensed by RNA PRRs [14, 15] but in turn possesses mechanisms to evade the innate immune response [21, 24, 25]. Here, we evaluate the role of cGAS during BTV infection, showing that BTV induces cytosolic DNA accumulation, which could possibly be caused by mitochondrial stress during infection. Also, in concordance with previous studies from members of our team in the context of dengue virus (DENV) and chikungunya virus (CHIKV) infections, our findings show that BTV infection leads to cGAS degradation through an autophagy-dependent mechanism mediated by the NS3 viral protein [11, 13].

These reports demonstrate that DENV and CHIKV can trigger cGAS activation by causing mtDNA accumulation in the cytosol due to mitochondria membrane damage during the infection [11, 13]. Other viruses, such as SARS-CoV-2, activate cGAS by inducing accumulation of cytoplasmic chromatin [40], proving that cGAS sensing is not only limited to DNA virus infection. In our study, we show that mitochondrial membrane potential decreases during BTV infection, which likely leads to the permeabilization of the mitochondrial membrane. We also describe the accumulation of DNA in the cytoplasm of BTV-infected cells concomitantly to the reduction in the labeling of the mitochondrial membrane transporter TOM20, which could be linked to the disruption of the mitochondrial membrane. Furthermore, this accumulation was not dependent on NS3 expression, since DNA was still detectable in the cytoplasm of BTV-8 ΔNS3-infected cells. Although the nature of this mis-localized DNA during BTV infection is yet to be identified, its accumulation in the cytosol can act as PAMP for cGAS activation [9].

Since cGAS is likely activated during BTV infection, we evaluated possible mechanisms by which the virus could evade PAMP sensing. We observed a drastic decrease of *Ifnα* and *Isg15* transcription levels in BTV-infected cells when cGAS was stimulated with DNA, indicating that DNA-induced IFN-I production was hindered by BTV infection. Intriguingly, we also observed a reduction of *Ifnα* and *Isg15* transcripts in cells treated with binary ethyleneimine (BEI)-inactivated BTV (iBTV), although this reduction was smaller than in BTV-infected cells. A possible explanation for the decrease of *Ifnα* and *Isg15* transcription in iBTV-treated cells is the presence of viral factors in structural components of the virion. BTV structural proteins VP3 and VP4 have been shown to interfere with IFN-I induction [23], yet the mechanism is not fully understood. At low concentrations, like we used in this study, BEI acts as an alkylation agent that blocks nucleic acid reading without altering protein structure [43]. This could indicate that although BTV cannot replicate when treated with BEI, VP3 and VP4 proteins could still interfere with IFN-I induction. However, a greater inhibition of IFN-I induction was observed in cells infected with non-inactivated BTV, indicating that viral replication is necessary to effectively interfere with IFN-I production.

The substantial decrease of IFN-I production in BTV-infected cells correlates with cGAS and STING degradation observed during infection. The degradation became more pronounced as virus concentration increased. Furthermore, cGAS inhibition significantly increased BTV replication, indicating that cGAS sensing of BTV, despite being an RNA virus, plays a relevant biological role in limiting viral growth, as has been shown with other RNA viruses [11, 13, 44]. It is therefore likely that BTV has evolved mechanisms to actively promote cGAS and STING degradation. Amongst BTV proteins, NS3 and NS4 have been described as IFN-I antagonists [21, 23], which is why we evaluated the possibility that these proteins could be responsible for cGAS and STING degradation. cGAS expression was not altered in the presence of BTV-NS4. Nonetheless, we did observe cGAS degradation in the presence of BTV-NS3 in a dose-dependent manner. Although we observed a decrease in STING expression during BTV infection, we were not able to determine the viral protein responsible for this degradation, as neither NS3 nor NS4 altered STING expression levels. This result is similar to the findings described for chikungunya virus, another RNA virus, which also induces cGAS degradation. In this case, whilst the capsid protein was identified as responsible for cGAS degradation, STING expression remained unaltered during infection, although viral protein nsP1 was discovered to interact with STING [13]. In the case of BTV, it could be possible that more than one viral protein is required for STING degradation. Further understanding of the interactions between BTV proteins could help better comprehend the mechanism behind STING degradation.

Apart from being the main viral protein involved in virion egress from the host cell [45], BTV-NS3 is also an interferon antagonist that avoids RNA sensing by blocking the RLR-dependent signaling pathway [23]. In this work, we demonstrate that BTV-NS3 is also responsible for evading cytosolic DNA sensing by degrading cGAS. This finding was confirmed by analyzing cGAS expression during infection with BTV-NS3 defective mutant (BTV-8 ΔNS3), which proved that NS3 viral protein is required for cGAS degradation. Taken together, these data expose the versatility of this viral protein to elude host sensing of infection. Indeed, BTV-NS3 deletion mutant based on an attenuated BTV has been generated as potential vaccine candidate, since it is less efficient in IFN response evasion and produces a strong immune response [46].

Besides being an IFN antagonist, BTV-NS3 is a transmembrane protein present in the plasma membrane that binds to cellular protein Tsg101 and with BTV capsid protein VP2, allowing virion transport to the cell surface [47, 48]. The ability of this protein to bind to both cellular and viral components is further confirmed in the present work as we detect a physical interaction between NS3 and cGAS. As we have described in previous work [24], NS3 is also capable of binding to STAT2, interfering with the IFN-I signaling pathway. This leads us to hypothesize that NS3 likely possesses binding domains that allows the protein to interact with multiple components of the IFN induction and signaling pathways as a mechanism to evade the innate immune response at multiple levels. In addition, we show that NS3-mediated cGAS degradation is reduced when the autophagy pathway is blocked by autophagy inhibitors MG-132 or BAF-A1, or by *ATG7* gene silencing. Thus, NS3 induces cGAS degradation through an autophagy-dependent mechanism. Furthermore, protein expression analysis revealed that Atg7 protein expression was increased and that LC3-II was accumulated in the presence of BTV-NS3 protein. During autophagy, Atg7 acts as an E-1 enzyme for ubiquitin-like proteins and is critical for phagophore formation [36, 49], and cytosolic LC3 (LC3-I) is modified to LC3-II, which is recruited for autophagosome formation [50]. The fact that in this study we observe an increase of Atg7 expression and LC3-II accumulation in the presence of BTV-NS3 supports the findings of previous studies that indicated that BTV induces autophagy to favor viral replication [51]. Moreover, virus-mediated autophagic degradation of cGAS has also been described for other RNA viruses, such as dengue and Chikungunya [11, 13].

**Fig. 8:**
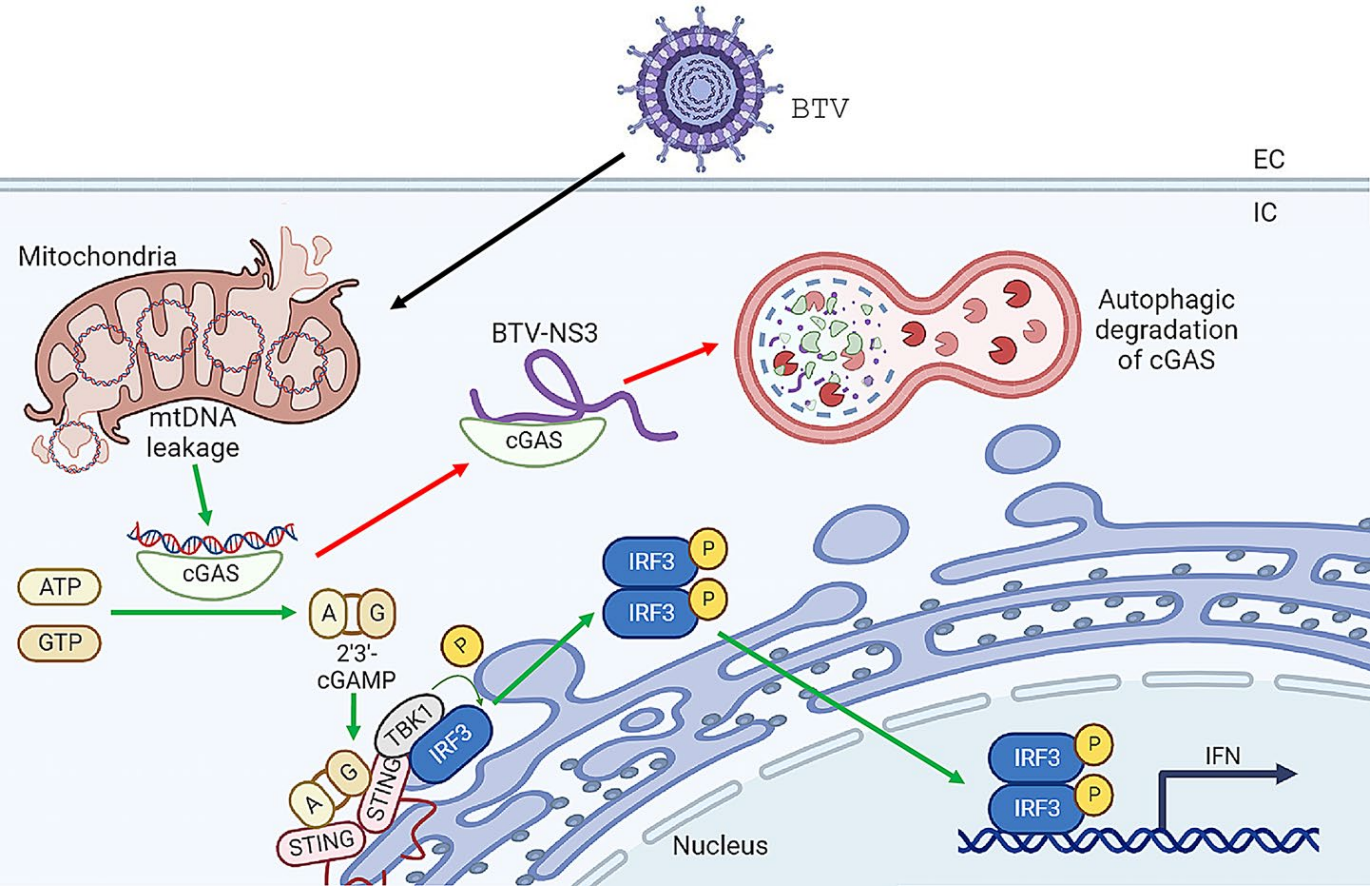
Schematic representation of cGAS degradation during BTV infection. Virus infection leads to a decrease in mitochondrial membrane potential, alterations in mitochondria morphology and accumulation of cytosolic DNA, potentially activating cGAS-STING pathway to induce IFN transcription to combat the infection. However, BTV is capable of degrading cGAS through an autophagy-dependent process mediated by BTV-NS3 protein, which binds to cGAS and induces its degradation as a mechanism to antagonize the IFN response

Our previous work revealed that BTV-NS3 is ubiquitinated in lysine residues 13 and 15, and that this ubiquitination is required for STAT2 degradation [24], hypothesizing a model in which NS3 acts as an adaptor that interacts with STAT2 and is ubiquitinated with Ub K63 chains, inducing the autophagic degradation of the complex. When we studied the interaction between NS3 and cGAS, we also evaluated the importance of NS3 ubiquitination in the degradation process. Western blot analysis revealed that non-ubiquitinated NS3 mutant did not effectively degrade cGAS and could not reduce IFN-I production to the same degree as wildtype NS3. This finding supports the hypothesis that NS3 acts as a scaffold protein that binds to cellular components, forming a protein complex tagged with ubiquitin chains that are sensed by the host cell for autophagic degradation, manifesting the versatility of this protein to antagonize the interferon response (Fig. 8). For this reason, it is likely that future studies will be able to find other cellular components with which BTV-NS3 protein interacts to evade the innate immune response.

It is now known that cGAS sensing pathway restricts both DNA and RNA viral infections. Here we identify for the first time an Orbivirus that directly antagonizes a DNA sensing pathway. In this work we demonstrate that although BTV could potentially activate DNA-sensing pathways, it possesses mechanisms to evade cytosolic DNA detection by the host cell.

## Electronic supplementary material

Below is the link to the electronic supplementary material.

Supplementary Material 1

Supplementary Material 2

Supplementary Material 3

## Data Availability

Data sharing is not applicable to this article as no datasets were generated or analyzed during the current study. All data generated or analyzed during this study are included in this article and are accessible from the corresponding author upon reasonable request.
