# Supplementary figures and images for: Double-stranded RNA orbivirus disrupts the DNA-sensing cGAS-sting axis to prevent type I IFN induction
Source: Cell Mol Life Sci. 2025 Jan 21;82(1):55. doi: 10.1007/s00018-025-05580-5 (PMC11751250; doi:10.1007/s00018-025-05580-5)

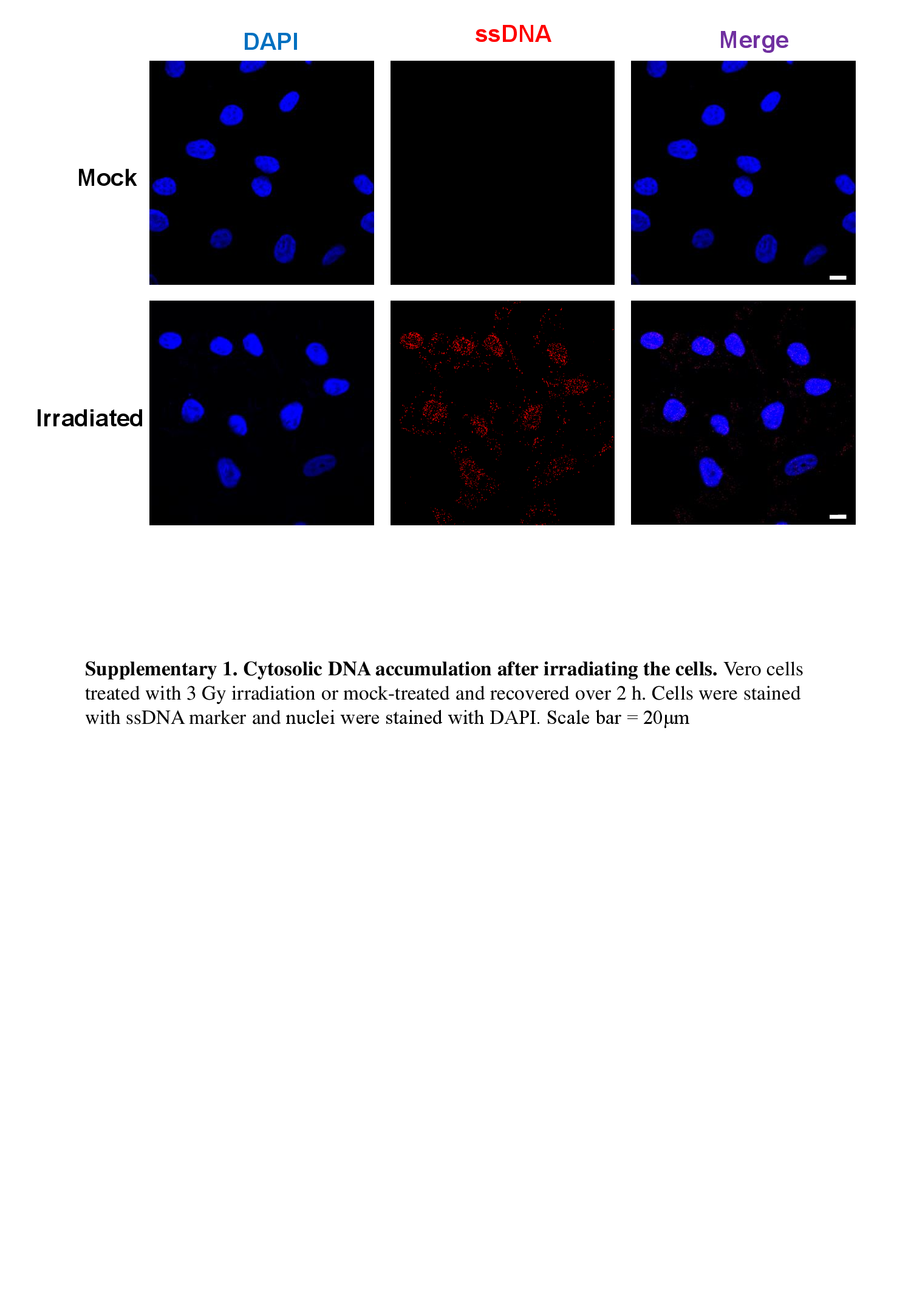

Supplement: Supplementary file 1 — Supplementary Material 1 [file 18_2025_5580_MOESM1_ESM.tiff]

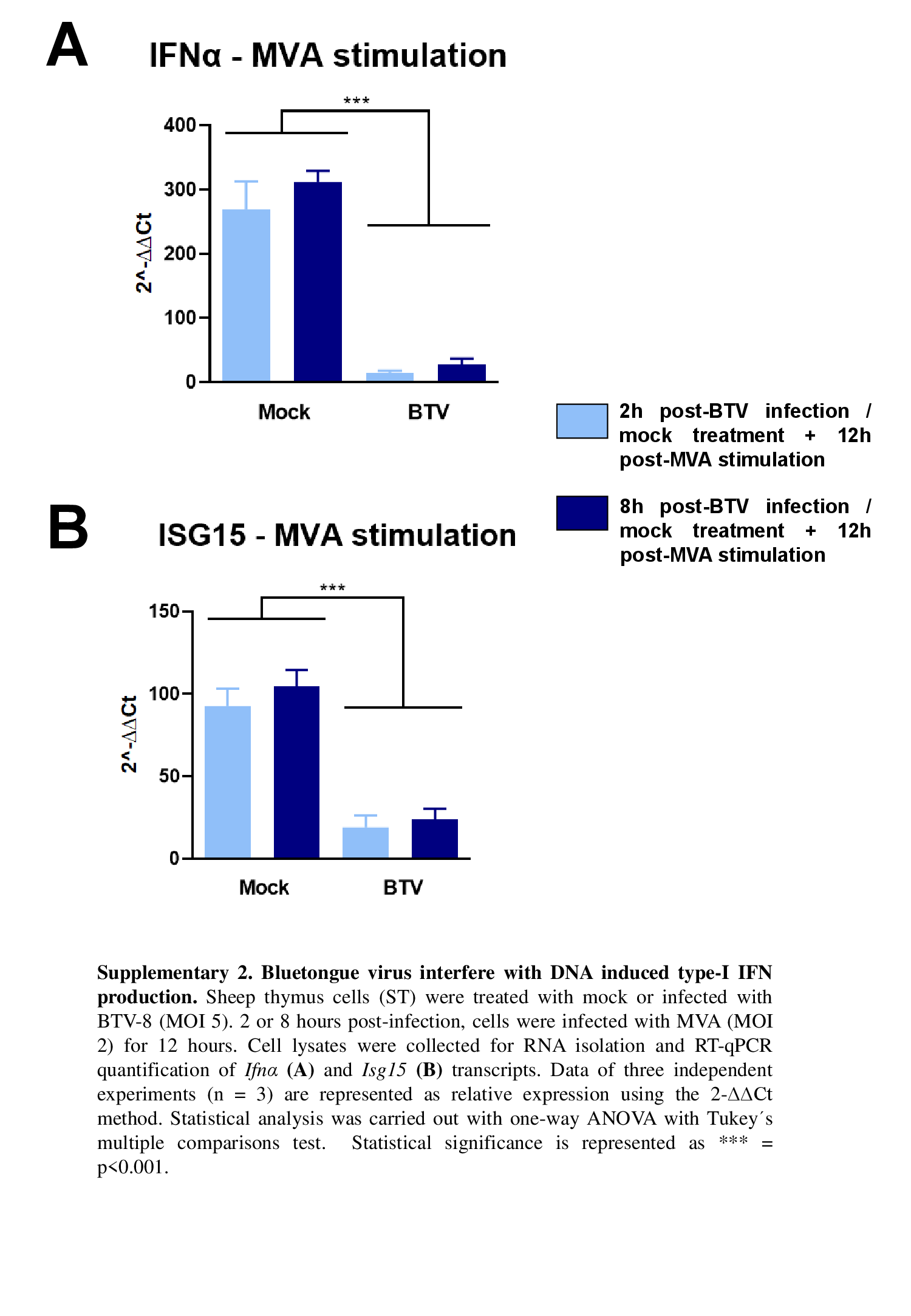

Supplement: Supplementary file 2 — Supplementary Material 2 [file 18_2025_5580_MOESM2_ESM.tiff]
